# Sarcomas of the breast: findings on mammography, ultrasound, and magnetic resonance imaging

**DOI:** 10.1590/0100-3984.2016.0141

**Published:** 2018

**Authors:** Renato Augusto Eidy Kiota Matsumoto, Su Jin Kim Hsieh, Luciano Fernandes Chala, Giselle Guedes Netto de Mello, Nestor de Barros

**Affiliations:** 1 Department of Radiology and Oncology, Faculdade de Medicina da Universidade de São Paulo (FMUSP), São Paulo, SP, Brazil.; 2 Grupo Fleury, São Paulo, SP, Brazil.

**Keywords:** Breast cancer, Sarcoma, Mammography, Ultrasonography, Magnetic resonance imaging

## Abstract

Sarcomas of the breast belong to a heterogeneous group of breast tumors of mesenchymal origin, without epithelial components. These tumors can be primary or secondary (after previous treatment for breast cancer), are rare, present aggressive behavior, and have a poor prognosis. They occur mainly in women between 45 and 50 years of age, with the exception of angiosarcomas, which can occur in younger patients. Clinically, breast sarcomas manifest as palpable, mobile, rapidly growing masses, without skin thickening, axillary lymphadenopathy, or nipple discharge. Although the imaging findings are non specific, they can be suggestive of sarcoma. For instance, a solitary mass showing rapid growth, with circumscribed or indistinct margins and, a complex (solid-cystic) or heterogeneous echotexture, without axillary lymph node involvement, can raise the suspicion of sarcoma. The treatment is not well established, because of the rarity and heterogeneity of this type of neoplasm. The principles of treatment for sarcoma of the breast have been addressed only in small cohort studies. In most cases, the treatment of choice is surgery without axillary lymphadenectomy.

## INTRODUCTION

Primary breast sarcomas constitute a rare group of nonepithelial tumors with aggressive behavior, originating from connective breast tissue^(^^1^^)^. They account for less than 1% of all breast malignancies and less than 5% of all soft tissue sarcomas^(^^2^^-^^4^^)^. Sarcomas occur predominantly in women, primarily in those between 45 and 50 years of age^(^^3^^)^. Angiosarcomas are an exception, being found in younger women, reportedly with a mean age of less than 40 years^(^^2^^)^. The main objective of this article is to describe the most common imaging findings and clinical aspects of sarcomas of the breast, on the basis of illustrative cases.

## CLINICAL ASPECTS

**Figure 1:**
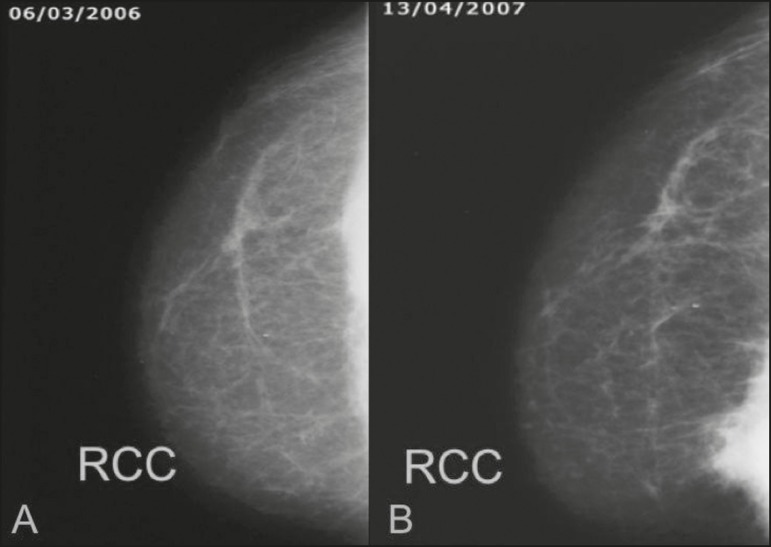
A 52-year old woman who previously underwent bilateral mastectomy for invasive ductal carcinoma. Right breast reconstruction with a transverse rectus abdominis muscle flap. The patient had undergone radiotherapy 12 years before. Mammogram showing an irregular, spiculated, hyperdense, 8-cm mass in the right breast.

The main clinical presentation of a sarcoma of the breast is that of a palpable, mobile, rapidly growing mass, without skin thickening, nipple discharge, or palpable axillary lymph nodes^(^^3^^,^^4^^)^. Sarcomas of the breast can form masses up to 30 cm in diameter, with a mean diameter of 3 cm^(^^3^^)^. Tumor size is an important prognostic factor, given that the overall survival rate is better for patients with tumors less than 5 cm in diameter^(^^3^^)^. The dissemination is mainly hematogenous, cells spreading to the lungs, bones, liver, and central nervous system, with only discreet lymphatic spread^(^^3^^,^^4^^)^. The main risk factors for developing sarcoma of the breast are previous radiotherapy (Figure 1) for thoracic neoplasms or Hodgkin lymphoma, and genetic syndromes, such as neurofibromatosis type 1 and Li-Fraumeni syndrome^(^^3^^)^. Some environmental factors are also associated with sarcoma of the breast, such as exposure to arsenic, herbicides, and immunosuppressive agents^(^^5^^)^. The most widely used staging system for sarcomas of the breast is that of the American Joint Committee on Cancer, which takes into consideration the histological grade and size of the tumor, together with the regional lymph node status and the presence or absence of distant metastases^(^^6^^)^.

## HISTOLOGY

Sarcomas of the breast are considered a heterogeneous group of malignant neoplasms of interlobular mesenchymal origin, the elements comprising the supporting stroma^(^^1^^,^^7^^)^. The most common subtypes are angiosarcoma, fibrosarcoma, and undifferentiated pleomorphic sarcoma^(^^2^^)^. The high incidence of angiosarcoma is related to prior radiotherapy for breast carcinoma and chronic lymphedema^(^^5^^)^. It is recommended that a core needle biopsy be performed, to provide a greater amount of material for histological analysis. However, there is some concern about the tumor spreading along the biopsy pathway and it is therefore important to plan the procedure well, in order to minimize this complication, and to excise the pathway during surgery^(^^8^^)^.

## IMAGING FINDINGS

### Mammography

**Figure 2:**
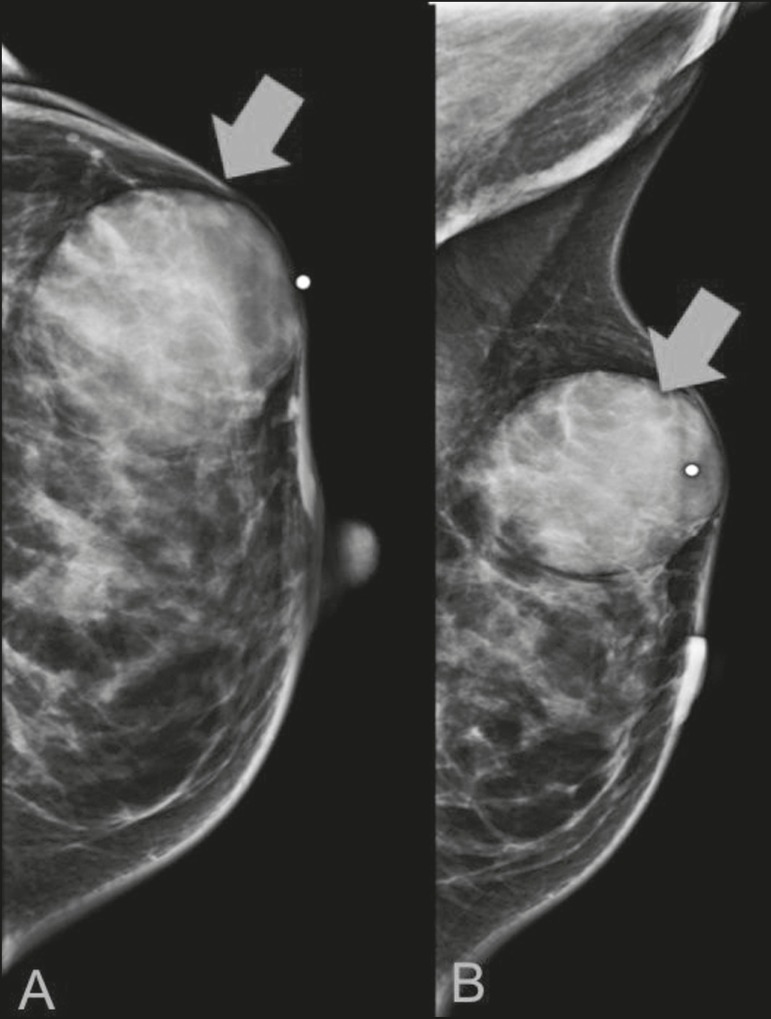
A 42-year-old woman with a palpable mass in the left breast (radiopaque marker). Mammogram showing a round, circumscribed, hyperdense mass, measuring 5.0 cm in diameter, in the upper outer quadrant of the left breast (arrows). Pathological diagnosis: fibrosarcoma.

**Figure 3:**
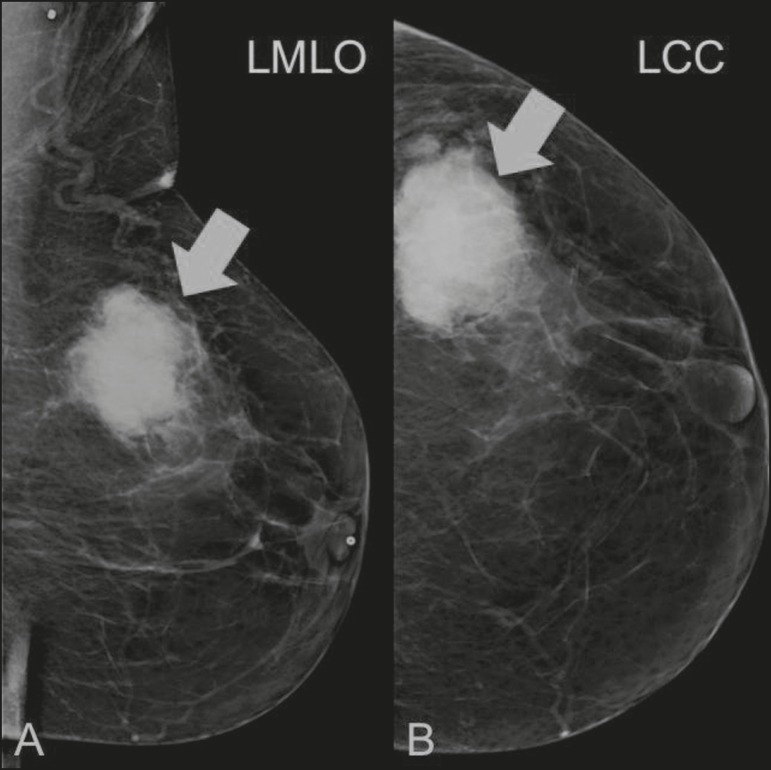
A 78-year-old woman, who underwent right mastectomy for invasive ductal carcinoma 30 years prior, with a palpable mass in the left breast. Mammogram showing an irregular hyperdense mass, with indistinct margins, in the upper outer quadrant of the left breast (arrows). Pathological diagnosis: leiomyosarcoma.

**Figure 4:**
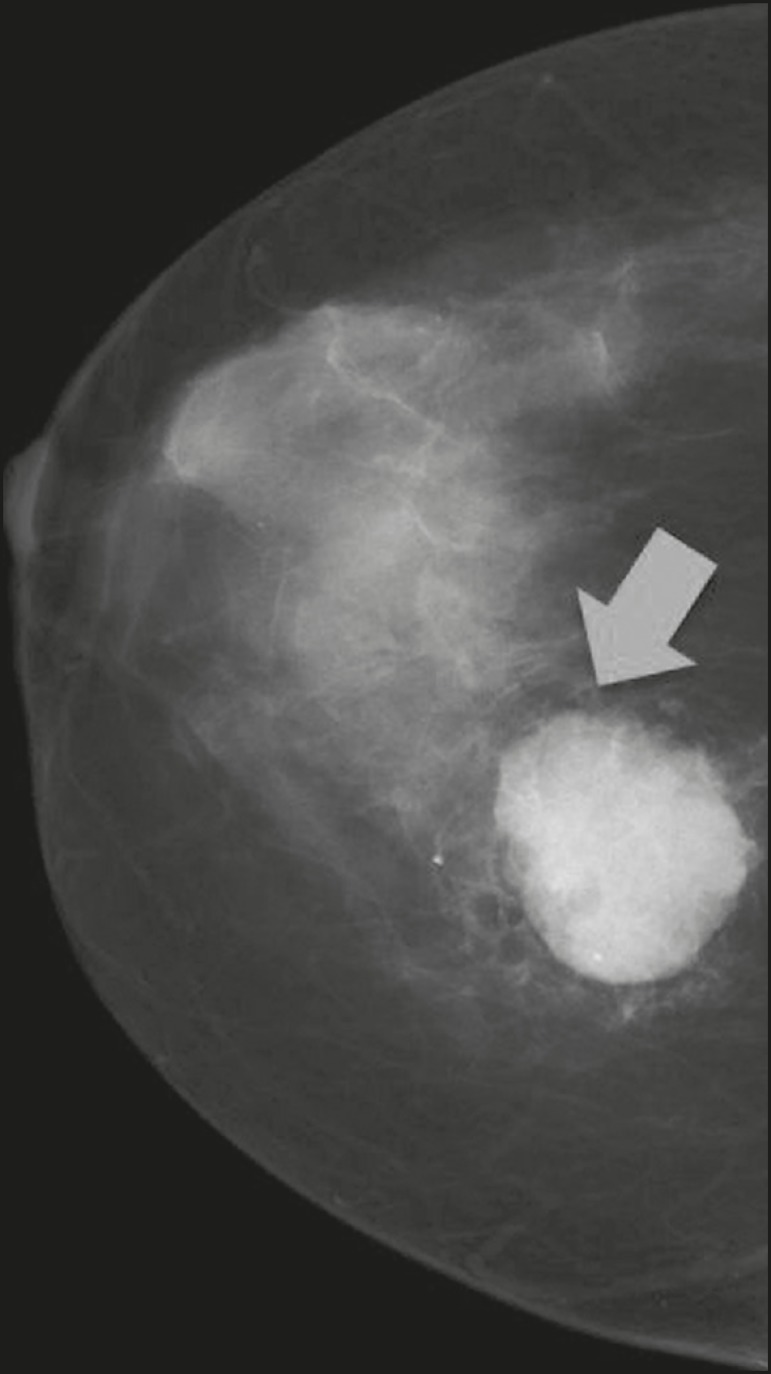
Mammogram of a 46-year-old woman with a palpable, high-density, oval, circumscribed, noncalcified mass in the lower inner quadrant of the right breast.

The most common mammographic finding is a single oval hyperdense mass with indistinct or circumscribed margins and no calcifications (Figure 2). Spiculated margins are rarely seen. The presence of indistinct margins (Figures 3 and 4) and the absence of calcifications are the most valuable mammographic features^(^^9^^)^. In one study of sarcomas of the breast, Surov et al. described 68% of the lesions as masses, reporting that 46% had a lobular morphology, 31% had a round morphology, and 77% had microlobulated margins^(^^9^^)^. The authors observed calcifications in 16% of the cases. Smith et al. used mammography to evaluate 16 primary breast sarcomas and reported that 14 (87.5%) presented as masses, 86% being oval and 64% having indistinct margins^(^^10^^)^.

### Ultrasound

**Figure 5.**
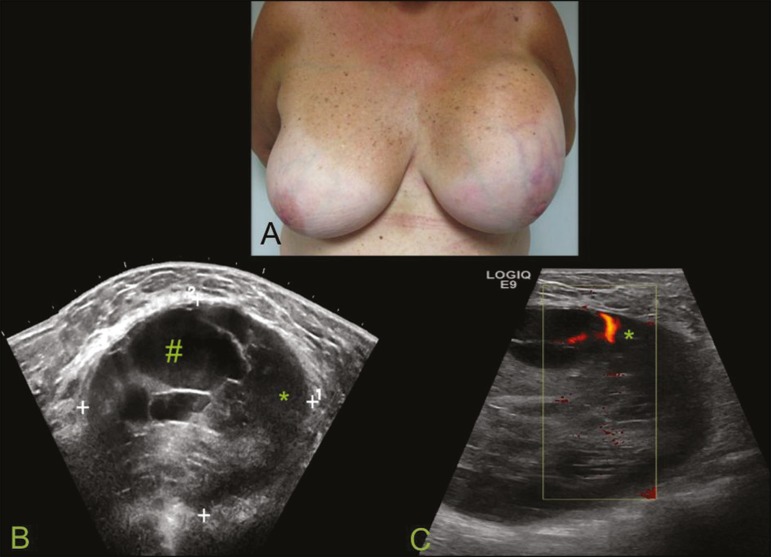
A: **A** 55-year-old woman with a palpable mass, showing rapid growth, in the left breast. **B,C:** Ultrasound showing a complex nodule with a solid component (asterisk) and a cystic component (#), together with an oval morphology and circumscribed margins, occupying the entire left breast. Pathological analysis: undifferentiated pleomorphic sarcoma.

**Figure 6:**
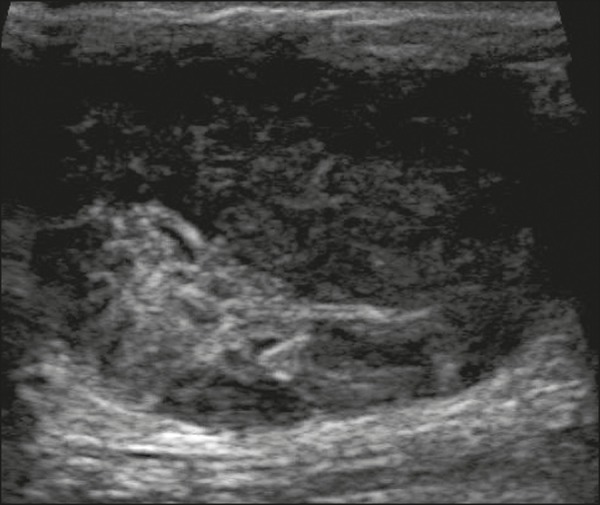
Ultrasound of a 48-year-old woman showing an oval, circumscribed, heterogeneous mass with a diameter of 4.5 cm, occupying the entire left breast. Pathological analysis: fibrosarcoma.

In the breast, ultrasound is better than mammography for evaluating the margins of a mass, for differentiating between solid and complex masses, for identifying and characterizing internal vascularization, and for guiding percutaneous procedures. On sonography, a sarcoma of the breast typically presents as an oval mass, with indistinct margins, a hypoechoic or complex echotexture, posterior acoustic shadowing, internal vascularization (on Doppler assessment), and no calcifications (Figures 5 and 6). It is uncommon to see skin thickening or suspicious axillary lymph nodes^(^^10^^)^.

### Magnetic resonance imaging

**Figure 7:**
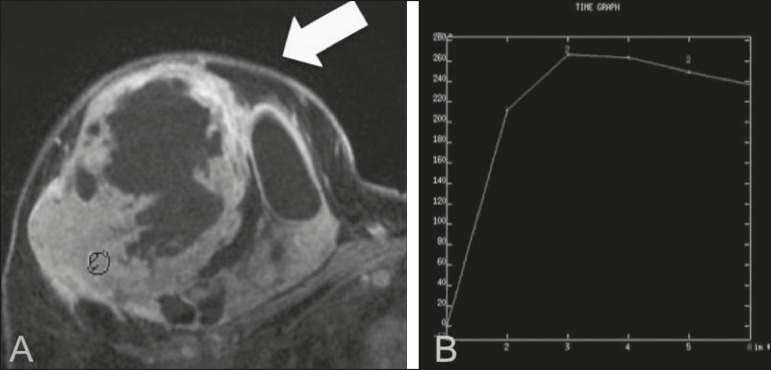
**A:** MRI showing a huge irregular mass with peripheral enhancement and necrotic areas. **B:** Kinetic study of the mass showing a plateau curve of contrast enhancement.

**Figure 8:**
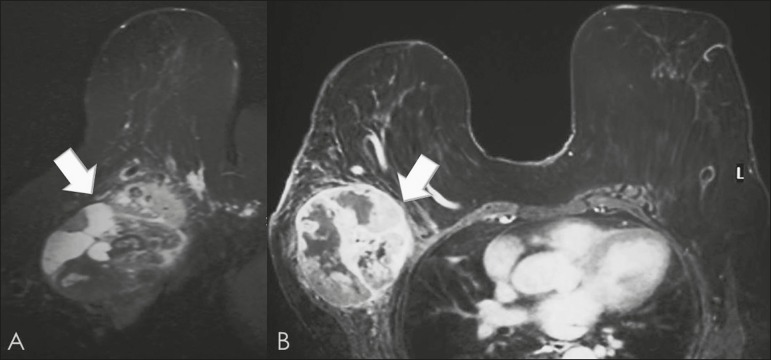
A 42-year-old woman with a palpable mass in the right breast. **A:** T2-weighted imaging showing an oval, circumscribed, complex mass, with cystic and solid areas (arrow). **B:** Contrast-enhanced T1-weighted imaging showing enhancement of the solid portion (arrow).

Like mammography and ultrasound, magnetic resonance imaging (MRI) of a breast sarcoma usually shows an oval mass, with irregular margins, a hypointense signal on T1-weighted imaging, a hyperintense signal on T2-weighted imaging, heterogeneous initial rapid enhancement, and washout or plateau curves in the late kinetic analysis, as depicted in Figures 7 and 8^(^^9^^)^. Smith et al.^(^^10^^)^ published the MRI findings of five patients with sarcomas. In four of those patients, the tumor presented as a single mass with variable enhancement. The most common morphology (in 75%) was an oval mass with irregular margins. All of the sarcomas showed a hyperintense signal on T2-weighted imaging, and half showed a hypointense signal on T1-weighted imaging, probably related to central necrosis^(^^10^^)^.

## SARCOMA OF THE MALE BREAST

**Figure 9:**
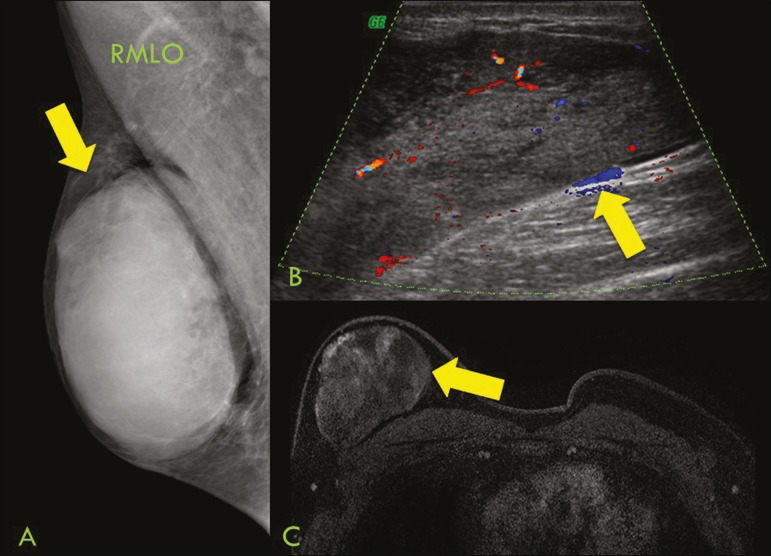
A 53-year-old man with a palpable, rapidly growing mass in the right breast. **A:** Mammography showing an oval, circumscribed, hyperdense mass, with faint calcifications, occupying the entire right breast (arrow). **B:** Ultrasound showing a solid, oval, circumscribed, heterogeneous mass (arrow) with internal vascularity on the Doppler flow study. **C:** Contrast-enhanced MRI (T1-weighted subtraction) showing the same mass, with circumscribed margins and heterogeneous enhancement (arrow). Histopathological diagnosis: liposarcoma.

Sarcomas are even rarer in male breasts than in women, occurring in 1.5% and 98.5% of cases, respectively^(^^5^^)^. The diagnostic algorithms do not differ for men. In male patients with a palpable mass, mammography and ultrasound are mandatory; percutaneous biopsy is also essential. The imaging characteristics are similar to those of sarcomas occurring in women, although there have been only sporadic reports of sarcomas of the breast in men (Figure 9).

## DIFFERENTIAL DIAGNOSES

### Phyllodes tumors

**Figure 10:**
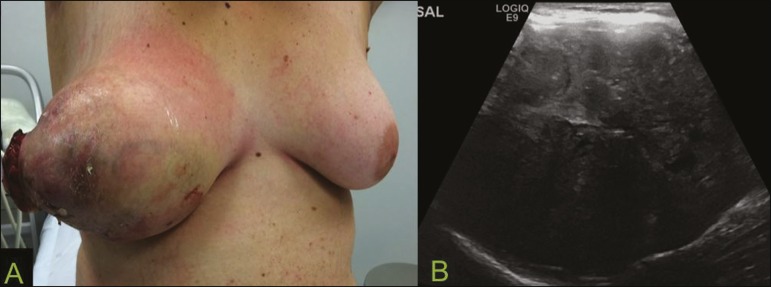
**A:** A 19-year-old woman with a rapidly growing mass in the right breast and no risk factors for breast carcinoma. **B:** Ultrasound showing an irregular, heterogeneous mass with indistinct margins, occupying all outer quadrants of the right breast. Histopathological diagnosis: phyllodes tumor.

Phyllodes tumors are usually excluded from breast sarcoma subgroups because they have benign epithelial components and malignant mesenchymal components^(^^5^^,^^7^^)^. The clinical presentation is that of a large, solitary, painless, mass. The patients are typically 10-20 years older than are those with typical fibroadenomas. On imaging, phyllodes tumors appear as solid, hypoechoic, oval, circumscribed masses, similar to fibroadenomas and sarcomas (Figure 10). On MRI, they show internal septa and liquid-filled spaces, with a hyperintense signal on T2-weighted imaging.

### Metaplastic carcinoma

Metaplastic tumors are special invasive breast carcinomas with squamous or spindle cells of epithelial origin. They present as round masses with indistinct margins on mammography and as solid or complex masses on ultrasound, similar to primary breast sarcomas. However, axillary involvement is more common in metaplastic carcinoma, because lymphatic spread is rare in sarcomas.

### Invasive ductal carcinoma not otherwise specified

The most common invasive breast carcinoma subtype, invasive ductal carcinoma not otherwise specified, presents with unique imaging features. Irregular, spiculated masses, with or without pleomorphic or linear calcifications, are the main findings that should raise suspicion of this differential diagnosis.

### Triple-negative carcinomas

Triple-negative carcinomas constitute a molecular sub group of invasive carcinomas of the breast lacking expression of estrogen and progesterone receptors, without overex pression of human epidermal growth factor receptor 2. On imaging, triple-negative carcinomas have classically been described as round, circumscribed masses, similar to sarcomas. However, more recently, they have been widely described as irregular masses, with ill-defined margins and no typical suspicious calcifications.

### Juvenile fibroadenoma

Juvenile fibroadenoma, also known as giant fibroadenoma, appears as a single mass in young women (10-20 years of age) and can be quite large (up to 15 cm in diameter). On imaging, juvenile fibroadenomas can be similar to sarcomas, usually circumscribed without axillary lymphadenopathy.

### Lymphoma

Breast lymphomas are extremely rare, accounting for less than 1% of all breast malignancies. That could be a consequence of the paucity of lymphoid tissue in the breast. The sonographic findings are similar to those of sarcomas, although indistinct margins can favor a diagnosis of a sarcoma

## TREATMENT PRINCIPLES AND PROGNOSIS

The rarity of sarcomas of the breast and the lack of well-established criteria for their diagnosis have resulted in considerable variation in the reported rates of overall and disease-free survival. The 5-year survival rate reported in the literature, ranges from 14% to 91%^(^^2^^,^^3^^)^. Factors that lead to a worse prognosis for a breast sarcoma include an angiosarcoma subtype, a diameter greater than 5 cm, a high histological grade, positive margins, and recurrence^(^^2^^)^. Surgery is the treatment of choice for sarcomas of the breast. The local therapy should be aggressive, with negative margins or radical mastectomy^(^^2^^)^. Resection of the axillary lymph nodes is not routinely recommended, except when nodal positivity is confirmed, because metastasis to lymph nodes is estimated to occur in only 5% of cases^(^^5^^)^. Although there is no consensus regarding the use of radiotherapy in sarcomas of the breast, it is recommended for high-grade or large tumors^(^^2^^)^.

## CONCLUSION

The correct diagnosis of a sarcoma of the breast is extremely relevant, because of its aggressive behavior and poor prognosis. The imaging findings are not pathognomonic, percutaneous biopsy always being required in order to make the definitive diagnosis. Nonetheless, some characteristics can alert the radiologist to the possibility of sarcoma in the differential diagnosis. The presence of a rapidly growing mass, with indistinct or circumscribed margins, a heterogeneous or complex echotexture, and the absence of axillary lymphadenopathy all favor a diagnosis of sarcoma.
